# Metatranscriptomic analysis uncovers microbial and immune signatures underlying COVID-19 severity

**DOI:** 10.1093/bib/bbaf631.015

**Published:** 2025-12-12

**Authors:** Sanjana Fatema Chowdhury, Md Murshed Hasan Sarkar, Syed Muktadir Al Sium, Md Salim Khan

**Affiliations:** Bangladesh Council of Scientific and Industrial Research (BCSIR), Dhaka 1205, Bangladesh; Bangladesh Council of Scientific and Industrial Research (BCSIR), Dhaka 1205, Bangladesh; Bangladesh Council of Scientific and Industrial Research (BCSIR), Dhaka 1205, Bangladesh; Bangladesh Council of Scientific and Industrial Research (BCSIR), Dhaka 1205, Bangladesh

## Abstract

**Background:**

Beyond viral infection alone, emerging evidence suggests that the respiratory microbiome plays a key role in influencing any disease severity and immune modulation (1,2). While genomic studies of SARS-CoV-2 are abundant, host-microbiome interactions at the transcriptomic level remain underexplored, particularly in diverse, underrepresented populations. This study investigated the metatranscriptomic profiles of nasopharyngeal samples from Bangladeshi cohorts, aiming to identify microbial signatures, antimicrobial resistance gene (ARG) activity, and host immune responses associated with variable COVID-19 outcomes.

**Methodology:**

Forty nasopharyngeal samples representing four groups-severe, mild, asymptomatic, and negative, were sequenced using the Illumina NextSeq550 platform. Reads underwent preprocessing, host genome subtraction, and taxonomic and functional annotation. The reads that mapped with human genome were used for host differential expression, while unmapped reads were subjected to microbial taxonomic and functional analyses. This approach minimized cross-alignment errors and enabled integrated assessment of microbial diversity, antimicrobial resistance gene expression, and host pathway responses.

Microbial diversity (α and β) was calculated using Shannon, Simpson, and Bray–Curtis metrics. ARG profiles were identified via functional annotation, while differential gene expression and pathway enrichment of host transcripts were analyzed with DESeq2 and KEGG/GO tools.

**Results:**

**Microbial diversity and taxonomic profiles:**

Alpha diversity analysis showed significant prokaryotic diversity across severity groups, with asymptomatic samples clustering separately in β-diversity (PCoA) for both prokaryotes and eukaryotes. Severe cases showed widespread eukaryotic diversity, consistent with opportunistic fungal colonization. Relative abundance profiling revealed enrichment of pathogenic and multidrug-resistant bacteria (e.g., *Acinetobacter*, *Pseudomonas*) in COVID-19 positive patients, alongside increased fungal dominance beyond SARS-CoV-2 infection.

**Functional activity and AMR signatures of the microbiome:**

Metatranscriptomic profiling indicated upregulation of translation machinery, ribosomal proteins, and cold shock proteins in asymptomatic patients, suggesting active microbial function with reduced immune triggering. In contrast, mild patients displayed upregulated viral gene expression (e.g., replicase polyprotein, spike protein), while severe cases showed elevated stress response proteins and β-lactamase activity. GO and KEGG enrichment highlighted functional divergence in metabolic and immune-modulatory pathways across groups.

ARG analysis revealed asymptomatic patients harbored a distinct resistome enriched in aminoglycoside-, trimethoprim-, and rifamycin-resistance genes. TEM1-D β-lactamase was consistently abundant across all groups, suggesting widespread resistance potential.

**Host gene expression and immune signaling:**

Differential expression analysis identified 23 upregulated immune genes in COVID-19 positive patients, including *IFIT1*, *IFIT2*, and *CXCL10*, associated with cytokine-mediated signaling and type I interferon response. Severe and mild cases showed strong pro-inflammatory signaling, while asymptomatic patients uniquely displayed downregulation of TLR4 and reduced cytokine expression (IL-4, IL-13), potentially explaining their lack of clinical symptoms. Pathway analysis confirmed modulation of cytokine-cytokine receptor and Toll-like receptor signaling pathways depending on disease severity.

**Conclusion:**

This study reveals distinct microbial and host transcriptional signatures across COVID-19 severity groups. Asymptomatic patients exhibited unique microbial diversity, enriched antimicrobial resistance genes, and reduced innate immune activation, particularly through downregulated TLR4 signaling. In contrast, severe cases were marked by increased pathogenic load, stress responses, and heightened pro-inflammatory cytokine activity. Together, these findings underscore the interplay between the respiratory microbiome, ARGs, and host immune responses in shaping COVID-19 outcomes. These insights highlight the potential of integrated metatranscriptomic approaches for understanding infectious disease heterogeneity, and emphasize the need for validation in larger, ethnically diverse cohorts with detailed clinical metadata.

**References:**

1. Chen J, Liu X, Liu W, Yang C, Jia R, Ke Y, Guo J, Jia L, Wang C, Chen Y. ‘Comparison of the respiratory tract microbiome in hospitalized COVID-19 patients with different disease severity.’ Journal of Medical Virology. 2022 Nov;94(11):5284–93.

2. Garcia-Nuñez M, Millares L, Pomares X, Ferrari R, Pérez-Brocal V, Gallego M, Espasa M, Moya A, Monsó E. ‘Severity-related changes of bronchial microbiome in chronic obstructive pulmonary disease.’ Journal of clinical microbiology. 2014 Dec;52(12):4217–23.

